# Association of Transferable Quinolone Resistance Determinant *qnrB19* with Extended-Spectrum **β**-Lactamases in *Salmonella* Give and *Salmonella* Heidelberg in Venezuela

**DOI:** 10.1155/2013/628185

**Published:** 2013-09-25

**Authors:** Fanny González, María Araque

**Affiliations:** Laboratorio de Microbiología Molecular, Departamento de Microbiología, Facultad de Farmacia y Bioanálisis, Universidad de Los Andes, Mérida 5101, Venezuela

## Abstract

Four nontyphoidal *Salmonella* strains with resistance to extended-spectrum cephalosporins and nonclassical quinolone resistance phenotype were studied. Two *S.* Give were isolated from pediatric patients with acute gastroenteritis, and two *S.* Heidelberg were recovered from raw chicken meat. Phenotypic characterization included antimicrobial susceptibility testing and detection of extended-spectrum **β**-lactamases (ESBLs) by the double-disc synergy method. The detection of quinolone resistance-determining regions (QRDR) of *gyrA, gyrB*, and *gyrC* genes, *bla*_ESBLs_ genes, and plasmid-mediated quinolone resistance (PMQR) determinants was carried out by molecular methods. Plasmid analysis included Southern blot and restriction patterns. Transferability of resistance genes was examined by transformation. *bla*_TEM-1_ + *bla*_SHV-12_ genes were detected in *S.* Give SG9611 and *bla*_TEM-1_ + *bla*_CTX-M-2_ in the other three strains: *S.* Give SG9811, *S.* Heidelberg SH7511, and SH7911. Regardless of origin and serovars, the *qnrB19* gene was detected in the 4 strains studied. All determinants of resistance were localized in plasmids and successfully transferred by transformation. This study highlights the circulation of *qnrB19* associated with *bla*_TEM-1_, *bla*_SHV-12_, and *bla*_CTX-M-2_ in *S.* Give and *S.* Heidelberg in Venezuela. The recognition of factors associated with increasing resistance and the study of the molecular mechanisms involved can lead to a more focused use of antimicrobial agents.

## 1. Introduction

Nontyphoidal *Salmonella* (NTS) are one of the major causes of foodborne infections related to the ingestion of contaminated animal food products in humans [1]. In most cases, these infections are confined to the gastrointestinal tract and are self-limiting. However, for immunocompromised and/or elderly patients, as well as for invasive or prolonged infections, antibiotic treatment is recommended [2]. Fluoroquinolones and extended-spectrum *β*-lactams are the first-choice agents for these cases but the increase of the multidrug resistance (MDR) *Salmonella* strains reduces the available treatment options [1–5].

The emergence of *Salmonella* spp. isolates that display resistance to extended-spectrum *β*-lactams is mediated by plasmids and is an increasing public health concern [3–5]. The resistance to fluoroquinolones is typically mediated by alterations in the target enzymes DNA gyrase and topoisomerase IV or changes in drug entry and efflux. Also, three plasmid-mediated mechanisms conferring decreased susceptibility to ciprofloxacin have been recently described: QepA efflux, Aac(6′)-Ib-cr aminoglycoside acetyltransferase, and QNR proteins (*qnrA, qnrB, qnrC, qnrD*, and *qnrS*) [6, 7]. The *qnr* determinants encode pentapeptide repeat proteins, which are thought to bind to DNA gyrase, protecting it from quinolones; *aac*(6′)-*lb-cr* encodes a variant aminoglycoside acetyltransferase that modifies those fluoroquinolones that have a piperazinyl moiety, such as ciprofloxacin and norfloxacin, while *qepA* encodes a major facilitator efflux pump [6–8].

Several studies have shown the coexistence of plasmid-mediated quinolone resistance (PMQR) determinants with extended-spectrum *β*-lactamases (ESBL) on the same plasmid or cross-species/genera transferability of the plasmids [6, 9]. Selective pressure exerted by fluoroquinolones may be a driving force leading to the emergence and spread of isolates that carry not only PMQR determinants but also ESBL. Thus, close linkage between different resistance determinants may lead to high prevalence of MDR *Salmonella* strains under antibiotic-specific selective pressure [4, 5, 8]. In fact, the emergence of the association between PMQR and ESBL is an issue that narrows the usage of valuable antibiotics in managing NTS infections [1, 5, 9].

Epidemiologic studies of more than 2,300 NTS strains from human cases and animals in North America, Europe, and Asia have reported the prevalence of *qnrA, qnrB, qnrS*, and *aac*(6′)-*lb-cr* genes in 0.2%, 1.0%, 2.4%, and 6.4%, respectively [6]. On the other hand, in a previous study carried out by our team from 2005 to 2007 and in 2008, we found a prevalence of *qnrB19* (4.7%) in 117 NTS strains isolated from feces of pediatric patients and raw chicken meat [10]. Nevertheless, molecular and epidemiologic information on mechanisms of resistance in *Salmonella enterica* in Venezuela is scarce [10, 11]. The aim of this study was to determine the coexistence of PMQR determinant *qnrB19* and ESBL in *S*. Give and *S*. Heidelberg isolates from clinical sample and raw chicken meat in Mérida, Venezuela.

## 2. Materials and Methods

### 2.1. Salmonella Strains

In 2011, four NTS strains expressing a remarkable type of multidrug resistance were studied. These strains were selected from a collection of the Molecular Microbiology Laboratory of the Pharmacy and Bioanalysis Faculty of The Andes University, Mérida, Venezuela, based on resistance to extended-spectrum cephalosporins and an unusual quinolone resistance phenotype with reduced susceptibility to ciprofloxacin and associated susceptibility to nalidixic acid. Two *S.* Give (SG9611 and SG9811) were isolated from stool specimens obtained from pediatric patients with acute gastroenteritis, and two *S*. Heidelberg (SH7511 and SH7911) were recovered from raw chicken meat.

### 2.2. Antimicrobial Susceptibility Testing

The resistance patterns were determined by the standard plate agar dilution method, according to the Clinical and Laboratory Standards Institute (CLSI) guidelines [12]. The antimicrobial agents tested included (Sigma-Aldrich, St Louis, MO): cefoxitin, cefotaxime, cefotaxime-clavulanic acid, ceftazidime, ceftazidime-clavulanic acid, aztreonam, imipenem, meropenem, ertapenem, nalidixic acid, ciprofloxacin, amikacin, gentamicin, and tobramycin. *E. coli* ATCC 25922 was always used for quality control purposes. The ESBL phenotype was detected by the double-disc synergy (DDS) method [12].

### 2.3. Determination of Antimicrobial Resistance Genes and Sequence Analysis

#### 2.3.1. Detection of Mutations in the Topoisomerase Genes *gyrA*, *gyrB*, and *parC*

PCR was used to amplify the quinolone resistance-determining regions (QRDR) of target genes to identify mutations in each isolate. DNA was prepared, and the QRDR of *gyrA*, *gyrB*, and *parC* were amplified with previously described primers and protocols [13].

#### 2.3.2. Detection of PMQR

Screening for *qnrA*, *qnrB*, *qnrS*, *qnrD*, *aac(6*′*)-Ib*, and *qepA* genes was carried out by multiplex and simplex PCR amplifications using a previously described method and specific primers [14, 15].

#### 2.3.3. Detection of *β*-Lactamase Genes

*bl* *a*_TEM_, *bla*_SHV_, and group *bla*_CTX-M_ genes were detected by PCR using previously described primers and protocols [16].

All amplification products were purified (PCR-Accuprep kit Bioneer), and their nucleotide sequencing was performed with the 3730XL genetic analyzer (Applied Biosystems, CA, USA). Nucleotide and amino acid sequence alignments were analyzed using the Basic Local Alignment Search Tool (BLAST) suite of programs (http://blast.ncbi.nlm.nih.gov/Blast.cgi).

### 2.4. Plasmid Analysis

Plasmid DNA was extracted using the QIAprep spin miniprep kit (Qiagen Inc., USA). The presence of *bla*_ESBLs_ and PMQR determinants was ensured using Southern blot analysis on nylon membranes as described for the dot blot hybridization [17]. These plasmids were analyzed by restriction mapping using the HindIII and PstI enzyme (Promega, Madison, WI, USA) to observe the restriction patterns, and the resulting fragment sizes were determined by gel electrophoresis.

### 2.5. Transformation Experiment

ESBL and Qnr-harboring plasmids were transferred into *E. coli* HB101 competent cells by electroporation (Eppendorf model 2510, Germany) using approximately 0.5 *μ*g of the plasmid DNA preparation under conditions recommended by manufacturer. Mueller Hinton agar supplemented with 8 *μ*g/mL cefotaxime and 0.06 *μ*g/mL ciprofloxacin was used for selection of transformants. The presence of *bla*_ESBLs_ and *qnr *genes in the transformants was confirmed by antimicrobial susceptibility and PCR assays. Results were compared with those of the corresponding donor strains.

## 3. Results

### 3.1. Antimicrobial Susceptibility Testing

MICs of selected antibiotic for *S*. Give and *S.* Heidelberg strains and their transformants are presented in Table 1. All strains were resistant to all tested extended-spectrum cephalosporins and monobactams. By using clavulanic acid, an 8- to 10-fold reduction of MICs of cefotaxime and ceftazidime was observed. A positive double-disk test also confirmed ESBL production. These strains remained fully susceptible to cefoxitin, carbapenems, and amikacin. *S*. Give SG9811 and *S*. Heidelberg SH7911 were resistant to gentamicin (32 *μ*g/mL and >64 *μ*g/mL, resp.) while *S*. Give SG9611 was resistant to tobramycin (16 *μ*g/mL).

ESBL and quinolone-resistance-harboring plasmids were successfully transferred by transformation from all parental strains to the recipient *E. coli* HB101 strain. Negligible differences were found among the MICs of the transformants and their *Salmonella* donors. Only *S*. Heidelberg SH7511 and SH7911 strains, showing a phenotype of intermediate susceptibility to gentamicin and tobramycin (8 *μ*g/mL), respectively, produced transformant cells (7511trf and 7911trf) clearly defined as resistant to gentamicin (16 *μ*g/mL) and sensitive to tobramycin (4 *μ*g/mL).

### 3.2. Plasmid Analysis

Plasmid purified from 9811trf, 7511trf and 7911trf of ~19 kb exhibited similar restriction pattern generated by single as well as double digestion HindIII and PstI, whereas p9611trf of ~13 kb had a unique restriction pattern. However, Southern blot experiments confirmed that *bla*_ESBL_ and *qnr* alleles were present in all isolated plasmids (data not shown).

### 3.3. Detection of *β*-Lactamases and *qnr* Genes

PCR amplification, using specific primers for *bla*_TEM_, *bla*_SHV_, and group *bla*_CTX-M_, and sequencing analysis, allowed us to identify *bla*_TEM-1_ + *bla*_SHV-12_ in *S*. Give SG9611 and *bla*_TEM-1_ + *bla*_CTX-M-2_ in the other three strains: *S*. Give SG9811, *S*. Heidelberg SH7511, and *S*. Heidelberg SH7911. Regardless of origin and serovars, the *qnrB* gene was detected in the 4 strains studied (Table 2). Sequence analysis of the amplification product revealed the *qnrB19* variant with a 99% degree of identity, corresponding to the nucleotide sequence reference GenBank accession no. EU432277. No mutations were identified in the QRDR of the *gyrA*, *gyrB*, and *parC* genes.

## 4. Discussion

Since 2007, we have been reporting the presence of NTS producing gastroenteritis in pediatric patients from urban areas and in asymptomatic children from indigenous communities in Venezuela [11, 18, 19]. In these studies, *Salmonella* strains remained susceptible to cephalosporins and fluoroquinolones. Subsequently, in 2010, *S*. Heidelberg and *S*. Enteritidis strains isolated from food showed production of ESBL not associated with resistance to fluoroquinolones [20]. Recently, we also reported the first description of the occurrence of the PMQR determinant *qnrB19* in serovars Havana, Give, Heidelberg, and Meleagridis in Mérida, Venezuela [10].

In this study, based on the patterns of resistance for *β*-lactam antibiotic and an unusual quinolone resistance phenotype with decreased susceptibility to ciprofloxacin (MIC: 0.5–1 *μ*g/mL) but still susceptible to nalidixic acid (MIC: 4–8 *μ*g/mL), we strongly suspect the presence of plasmids harboring *bla*_ESBLs_ and *qnr* genes in two different *Salmonella* serovars from clinical sample of pediatric patients and raw chicken meat.

Amplification and sequencing of *bla*_ESBLs_ and *qnr* genes revealed the presence of *bla*_TEM-1_ + *bla*_SHV-12_ in *S*. Give SG9611 and *bla*_TEM-1_ + *bla*_CTX-M-2_ in other three strains: *S*. Give SG9811,* S*. Heidelberg SH7511, and SH7911, associated in all cases with the *qnrB19* gene as the only determinant responsible for the quinolone phenotype.

The mechanism of the Qnr protective effect is not completely understood [6]. However, it has been proposed that the Qnr proteins interact with DNA gyrase and topoisomerase IV, hindering the action of the quinolones and minimizing their inhibitory effect, while isolates with a *qnr* gene may be less likely to develop topoisomerase mutations than other strains [6–8]. The results of this study agree with these observations. The QRDS of the gyrase and topoisomerase IV genes were screened for the most commonly occurring mutations, but no resistance-associated substitutions were detected.

Although there are few studies reporting the prevalence of *qnr* genes in *Salmonella* isolates from South American countries, alleles belonging to the *qnrB* are the most frequently detected in the *Enterobacteriaceae* family [1, 6], in accordance with the results obtained in this study.

Plasmids with *qnr* genes have been found to cotransfer TEM, SHV, and CTX-M genes. Particularly, the association between QnrB-like determinants and SHV-12, CTX-M-9, CTX-M-14, and CTX-M-15 has been reported in NTS strains [1, 4, 6, 7]. In this study, *qnrB19* and *bla*_TEM-1_, *bla*_SHV-12_, and/or *bla*_CTX-M-2_ genes located in plasmids were successfully transferred by transformation to susceptible *E. coli* recipient. Also, the resistance to gentamicin and tobramycin was cotransferred. It is recognized that insertion sequences (IS), as IS*CR1* or IS*Ecp1* associated with *qnrB* genes, provide a putative promoter region for the expression of genes encoding resistance to aminoglycosides, trimethoprim, chloramphenicol, and *β*-lactams [6, 21]. Additionally, the coexistence between *qnr* and aminoglycosides was expected because *qnrB* genes are frequently located in integrons [6, 9]. Regardless that all the four plasmids purified from transformant cells possessed *bla*_TEM-1_, *bla*_SHV-12_ or *bla*_CTX-M-2_, and *qnrB19*, only one of these (p9611trf) exhibited differences of size (~13 kb) and a particular restriction pattern. It is possible that the organization of diverse genetic elements that participate in mobilization, transposition, recombination, and resolution, as well as deletion and insertion of DNA, generates variations in size and diversity of plasmid structures.

According to the chronology of isolation and origin of the strains studied here, we believe that the source of the *qnr* gene might not be directly related to the use of quinolone, since these drugs are not prescribed in pediatrics patient, but possibly a coselection of the *qnr*-harboring plasmids could be related to a linkage with other resistance genes carried on the same plasmids (*bla*_ESBLs_). Moreover, fluoroquinolones are widely used in veterinary medicine and poultry production, and *qnr*-positive NTS isolates could be selected and transmitted to humans through the food chain [22].

Based on several reports, the use of fluoroquinolones in infections caused by *Salmonella* isolates with reduced susceptibility may lead to treatment failures and promote the selection of bacteria with higher-level resistance to quinolone, thereby fostering the effect of others mechanisms of resistance as mutations [5, 7, 8, 23].

The real prevalence of PMQR is usually underestimated as there are no reliable phenotypic methods to detect their presence [4]. Also, clinical isolates harboring PMQR determinants did not show any significant change in their MICs when compared with isolates susceptible to nalidixic acid and negative for presence of *qnr* genes. Hence, it is important to revise fluoroquinolones susceptibility testing practice, since strains with similar phenotype as those described in this study might be undetected in clinical laboratories using traditional phenotypic methods.

While prevalence data from an international survey that included 13 European countries identified the *qnrS* gene in a *Salmonella sp*. collection from human clinical sample and the *qnrD* gene in NTS strains of eight different serovars isolated from animal sources [24], and a Danish study reported the coexistence of *bla*_CTX-M-15_ and *bla*_SHV-12_ genes in a single plasmid associated with *qnrB2* genes in *S*. Concord and *S*. Senftenberg isolated from human patients [1], our results showed the presence of *qnrB19* associated with *bla*_TEM-1_, *bla*_SHV-12_, and *bla*_CTX-M-2_ in *S.* Give and *S*. Heidelberg from pediatric patients and raw chicken meat. Nevertheless, prevalence data should be interpreted with caution, since selection criteria for strains included in surveys could potentially bias the results because many studies have been performed with isolates collected over a short period of time, in the context of an outbreak or strains that are resistant to various antimicrobials agents [6, 22].

Unfortunately, data on the occurrence of ESBL associated with PMQR in NTS strains from South America are scarce. To date, only in Brazil the presence of a *qnrA1* in 100 isolates of *S*. Enteritidis and *qnrB19* in one strain of *S*. Corvallis from poultry origin has been described [25].

Although this study includes few NTS isolates, we could prove the association of transferable quinolone resistance determinant *qnrB19* with extended-spectrum *β*-lactamases in *S.* Give and *S.* Heidelberg in Venezuela. This represents a starting point for the development of surveillance programs aimed at the detection of ESBL and PMQR.

## 5. Conclusion

This study highlights the circulation of PMQR determinant *qnrB19* associated with *bla*_TEM-1_, *bla*_SHV-12_, and *bla*_CTX-M-2_ in *S.* Give and *S.* Heidelberg from pediatric patients and raw chicken meat in Venezuela. The emergence of the combination of PMQR genes with ESBLs may contribute to the appearance of MDR *Salmonella* and jeopardize the usage of valuable antibiotics. We hope that recognition of factors associated with increasing resistance and the study of the molecular mechanisms involved can lead to a more focused use of antimicrobial agents which, in turn, will reduce the selection and spread of PMQR determinants and/or ESBL-producing enteropathogens.

## Figures and Tables

**Table 1:** Antibiotic susceptibility of *qnrB* positive and ESBL-producing *S*. Give and *S*. Heidelberg isolates and their transformants, recipient *E. coli* HB101, and quality control *E. coli* ATCC 25922.

Antibiotics tested	MIC (mg/liter) for									
	*S.* Give		*E. coli* transformants		*S*. Heidelberg		*E. coli* transformants		*E. coli *	
	SG9611	SG9811	9611trf	9811trf	SH7511	SH7911	7511trf	7911trf	HB101	ATCC 25922
Cefoxitin	8	8	4	8	8	8	8	8	2	2
Cefotaxime	4	128	8	128	>256	>256	256	128	0.064	0.125
CTX/CLA	0.125	0.25	0.25	0.25	1	0.5	0.25	0.25	0.125	0.125
Ceftazidime	64	32	64	32	32	32	32	32	0.25	0.125
CAZ/CLA	0.25	0.125	0.25	0.25	0.25	0.25	0.125	0.125	0.25	0.125
Aztreonam	16	16	16	32	16	16	16	16	0.25	0.25
Ertapenem	0.064	0.064	0.064	0.064	0.064	0.064	0.032	0.064	0.064	0.032
Imipenem	0.064	0.064	0.064	0.064	0.064	0.064	0.064	0.064	0.032	0.032
Meropenem	0.064	0.032	0.064	0.064	0.064	0.064	0.064	0.064	0.032	0.032
Ciprofloxacin	1	1	1	1	1	1	1	2	0.125	0.015
Nalidixic Acid	8	4	8	4	4	8	4	8	2	1
Amikacin	4	2	2	2	8	2	8	8	0.25	0.25
Gentamicin	4	32	2	32	8	>64	16	64	0.5	1
Tobramycin	16	4	16	2	4	8	2	4	0.5	0.25

Other test DDST	+	+	+	+	+	+	+	+	−	−

CTX/CLA: cefotaxime/clavulanic acid; CAZ/CLA: ceftazidime/clavulanic acid; DDST: double disk synergy test.

**Table 2:** Phenotypic and genotypic characteristics of *S*. Give and *S*. Heidelberg isolates and their transformants (trf).

Strain number	Sample	Serovar	Resistance pattern	Plasmid size (kb)^a^	*β*-lactamase type	*qnr *gene
SG9611	Human	Give	ESBL+, CIP, TOB	13	*bl* *a*_TEM-1_ + *bla*_SHV-12_	*qnrB19*
9611trf			ESBL+, CIP, TOB	13	*bl* *a*_TEM-1_ + *bla*_SHV-12_	*qnrB19*
SG9811	Human	Give	ESBL+, CIP, GEN	19	*bl* *a*_TEM-1_ + *bla*_CTX-M-2_	*qnrB19*
9811trf			ESBL+, CIP, GEN	19	*bl* *a*_TEM-1_ + *bla*_CTX-M-2_	*qnrB19*
SH7511	Chicken	Heidelberg	ESBL+, CIP, GEN_I_	19	*bl* *a*_TEM-1_ + *bla*_CTX-M-2_	*qnrB19*
7511trf			ESBL+, CIP, GEN	19	*bl* *a*_TEM-1_ + *bla*_CTX-M-2_	*qnrB19*
SH7911	Chicken	Heidelberg	ESBL+, CIP, GEN, TOB_I_	19	*bl* *a*_TEM-1_ + *bla*_CTX-M-2_	*qnrB19*
7911trf			ESBL+, CIP, GEN	19	*bl* *a*_TEM-1_ + *bla*_CTX-M-2_	*qnrB19*

^a^Estimated; ESBL+: extended-spectrum *β*-lactamase positive; CIP: ciprofloxacin; GEN: gentamicin; GEN_I_: intermediate sensibility to gentamicin; TOB: tobramycin; TOB_I_: intermediate sensibility to tobramycin.
